# Metabolic activity of mature biofilms of *Mycobacterium tuberculosis* and other non-tuberculous mycobacteria

**DOI:** 10.1038/s41598-017-10019-4

**Published:** 2017-08-23

**Authors:** Anna Solokhina, David Brückner, Gernot Bonkat, Olivier Braissant

**Affiliations:** 1 Center of Biomechanics & Biocalorimetry, University Basel, Gewerbestr. 14, CH-4123 Allschwil, Switzerland; 2 F. Hoffmann – La Roche, Ltd., Sterile Drug Product Manufacturing, Wurmisweg, CH-4303 Kaiseraugst, Switzerland; 3 Alta Uro AG, Centralbahnplatz 6, CH-4051 Basel, Switzerland

## Abstract

Mycobacteria are classified into two groups, fast- and slow-growing. Often, fast-growing mycobacteria are assumed to have a higher metabolic activity than their slower counterparts, but in mature biofilms this assumption might not be correct. Indeed, when measuring the metabolic activity of mycobacterial biofilms with two independent non-invasive techniques (isothermal microcalorimetry and tunable diode laser absorption spectrometry), mature biofilms of slow- and fast-growing species appeared more alike than expected. Metabolic heat production rate was 2298 ± 181 µW for *M. smegmatis* and 792 ± 81 µW for *M. phlei*, while *M. tuberculosis* and *M. bovis* metabolic heat production rates were between these values. These small differences were further confirmed by similar oxygen consumption rates (3.3 ± 0.2 nMole/s and 1.7 ± 0.3 nMole/s for *M. smegmatis* and *M. tuberculosis*, respectively). These data suggest that the metabolic potential of slow-growing mycobacterial biofilms has been underestimated, particularly for pathogenic species.

## Introduction

Since its discovery in 1882, there has been significant improvement in our understanding of *Mycobacterium tuberculosis*. However, this pathogen remains able to escape different treatments using various mechanisms^1, 2^. For *M. tuberculosis* (and other non-tuberculous mycobacteria (NTM)) both drug resistance^3, 4^ and drug persistence^5, 6^ are major issues. Drug resistance is linked to several genetic determinants, while drug persistence is the survival of a microbial subpopulation from the lethal effect of a drug. This subpopulation might later resume growth and lead to a persistent infection. This allows *M. tuberculosis* or other mycobacteria such as *M. avium* to persist for years in the human body, sometimes without detection^7^. Unlike resistance, however, persistence is a non-heritable phenotype^8^. There are many mechanisms involved in drug persistence, and a lower growth rate has often been correlated with drug persistence.

Drug resistance and persistence are also linked to biofilm formation. Indeed, *M. tuberculosis*, other mycobacteria, and many other pathogens can form biofilms^8–13^. In mycobacteria, stress and quorum sensing lead to the formation of a biofilm and persistent cells^11–13^. Animal models have shown that *M. tuberculosis* is often found in extracellular space^14^. Similarly, *M. tuberculosis* biofilms (or biofilm-like structures) are observed in primary lesion residual necrosis and in coating cavities^15, 16^. Such biofilm formation by *M. tuberculosis* and other mycobacteria has been shown to reduce their sensitivity to antimycobacterial agents^17, 18^. The biofilm architecture and the extracellular polymeric substance matrix limit drug diffusion, protecting individual cells^19, 20^.

Many researches have pointed to the lower activity of cells within biofilm as a potential mechanism for poor drug efficacy^6, 21^. However, across studies the term “activity” is often vaguely defined, and “low activity” usually refers to the fact that cell growth and multiplication are no longer observed and cells are expected to be dormant. In recent literature, persistence has been defined as an actively maintained state (i.e., a non-growing but metabolically active form)^7, 8, 22^. To the best of our knowledge, the metabolic activity of *M. tuberculosis* biofilms has never been determined in this context. Thus, microbiologists often assume that fast-growing mycobacteria such as *M. phlei* or *M. smegmatis* have a higher metabolic activity compared to their slow-growing counterparts such as *M. tuberculosis* and *M. bovis*. Unfortunately, no studies that focus on the metabolic activity of mycobacteria in mature biofilms exist to support this belief with direct evidence. Such knowledge about the metabolic activity of mature biofilms has critical implications for understanding *M. tuberculosis* biofilm physiology (including extra-pulmonary cases), drug development, and ultimately patient care.

There are several methods to assess metabolic activity, including reduction of tetrazolium salts (TTC, MTT, CTC) or resazurin (to assess the activity of reductases from the respiratory chain), use of GFP labeled microorganisms, or microautoradiography (MAR). For biofilm analysis, many of these techniques have shown strong technical limitations^23^ either because of their destructive nature, or because they cannot be applied to thick biofilms. The metabolic activity of biofilms can be measured by isothermal microcalorimetry (IMC)^24, 25^, a label-free technique that allows precise measurements in conventional, solid, and opaque media^26^. Heat production rate is directly linked to the overall metabolism (substrate consumption and byproduct release^27^) as shown by the glycerol respiration equation (Equation 1).

1 $$2{{\rm{C}}}_{3}{{\rm{H}}}_{8}{{\rm{O}}}_{3}+7{{\rm{O}}}_{2}- > 6{{\rm{CO}}}_{2}+8{{\rm{H}}}_{2}{\rm{O}}\quad \quad ({\rm{\Delta }}{\rm{H}}=-1655.2\,{\rm{KJ}}/{\rm{mol}})$$

IMC also allows for the focused study of mature biofilms grown on a solid medium at the surface of nylon membranes. Using this approach avoids the presence of planktonic cells, as very few will exist on such a membrane, enabling the study of a mature undisturbed biofilm. In addition to IMC, Tunable Diode Laser Absorption Spectroscopy (TDLAS) also allows non-invasive measurement of oxygen and carbon dioxide concentrations in the headspace to determine biofilm respiration rate^28, 29^. Most mycobacteria are considered strictly aerobic, consuming oxygen and releasing carbon dioxide (Equation 1), so TDLAS is therefore well suited to gather metabolic information. Both IMC and TDLAS provide independent measurements of biofilm metabolism that serve as a proxy for overall metabolic activity.

In this study, the metabolic activity of mature mycobacterial biofilms was investigated using model mycobacteria grown on membranes: *M. smegmatis* (DSM 43465), *M. phlei* (DSM 43239), *M. bovis* (DSM 43990) and *M. tuberculosis H37Ra* (ATCC 25177, an avirulent strain). We compared slow- and fast-growing mycobacterial biofilms, as the importance of growth rate and the presence of non-growing but metabolically active cells in these biofilms is emphasized in literature^7, 8, 22^.

## Material and Methods

### Microorganisms, growth conditions, and biofilm formation

**Figure 1:**
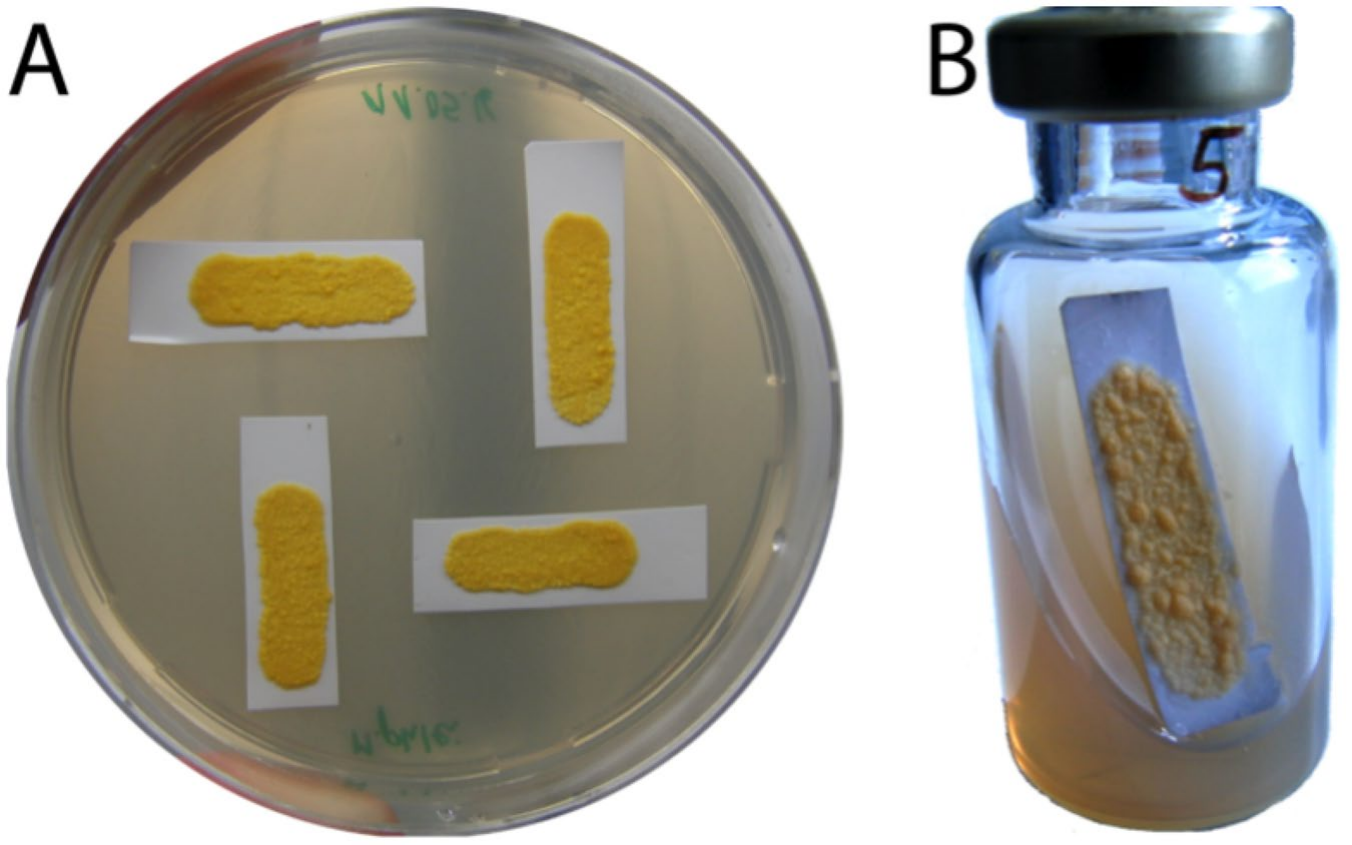
Picture of sample *M. phlei* biofilms prepared on 7H9 plates (**A**) and *M. phlei* biofilm prepared in a 20-ml calorimetric vial used for TDLAS measurements (**B**). Note that biofilms not reaching the required surface (at least 200 mm^2^) were not used for the experiment.

Mycobacteria were maintained frozen in Middlebrook 7H9 medium (Sigma-Aldrich, Buchs, Switzerland) supplemented with 25% glycerol. *Mycobacterium* strains were cultured in Middlebrook 7H9 medium supplemented with 10% OADC (Chemie Brunschwig AG, Basel, Switzerland). These cultures were incubated at 37 °C on a roller mixer until optical density (OD_600_) reached 0.25–0.5 (between 2 and 3 days for *M. smegmatis* and *M. phlei*, and up to 14 days for *M. tuberculosis* and *M. bovis*). Using a sterile 10 µl inoculating loop, liquid cultures were spread over sterile nylon filters cut to fit microcalorimetric vials. *Mycobacterium* biofilms formed on these filters were passed to fresh agarized 7H9 medium supplemented with 10% OADC until mature (no changes in appearance and size were observed) (Fig. 1A). Mature biofilms formed after 2 weeks for *M. smegmatis* and 3–4 weeks for *M. tuberculosis*. All biofilms had a surface area of at least 200 mm^2^. All materials and media used for the experiments were sterilized by autoclaving for 20 minutes at 121 °C.

### Metabolic heat production measurements

Metabolic heat production of *M. smegmatis* (DSM 43435), *M. phlei* (DSM 43239)*, M. bovis* (DSM 43990) and *M. tuberculosis* H37Ra (ATCC 25177, an avirulent strain) was monitored in an isothermal microcalorimeter (TAM Air, Waters/TA Instruments, USA). The microcalorimeter was equilibrated to 37 °C ± 0.01 for at least 24 hours and all measurements were performed at this temperature. The calorimetric ampoules (20 ml) were prepared with slanted solid 7H9 medium and mature biofilms were placed inside (Fig. 1B). The ampoules were sealed and introduced to the TAM Air, where heat production rates were monitored continuously until the heat-flow signal returned to baseline, indicating no further detectable metabolic heat production. For the biofilms measured in our study, heat-flows were measured up to 35 hours until baseline signals were reached again. Data were collected as ASCII files with a sampling frequency of 1 data point per minute.

### Headspace O_2_ and CO_2_ monitoring

Mature *M. smegmatis and M. tuberculosis* biofilms were further tested for oxygen and carbon dioxide concentration changes in sealed vial headspaces using a TDLAS spectrometer (Lighthouse Instruments, Charlottesville, VA, USA). Measurements were performed as previously described^29, 30^. The sample set used for this measurement was prepared similarly to those used for the calorimetric measurements. Devices were preheated for 30 minutes and calibrated to certified standards (0%, 2%, 4%, 8%, and 20%). Gas concentrations were measured at 762 nm (O_2_) and 2000 nm (CO_2_). Data was recorded directly as a spreadsheet and later converted into an ASCII file.

### Statistical analyses

All experiments were performed with at least 4 replicates. Data were analyzed using R statistical tools^31^ and the grofit package^32^ as described previously^33^. For IMC data, the maximum heat production rate (P_max_) was determined and heat-flow curves were integrated to obtain sigmoidal profiles (heat over time curves). Heat growth rate (usually used as a proxy for the growth rate - μ_Q_), and lag phase (λ) were determined by fitting the Gompertz model to these integrated data. For TDLAS, oxygen consumption and carbon dioxide production rates were estimated using a linear regression over the first 10 hours of measurements. Agreement between the techniques was assessed using Equation 1 to recalculate P_10_ (average heat flow over the first 10 hours) from oxygen consumption rate and carbon dioxide production rate. Comparison between the calculated parameters (μ_Q_, λ, time to peak, P_max_, P_10_) was performed using the Wilcoxon test after non-normality of the data was shown by the Shapiro Wilk test. In the case of multiple comparisons, the Dunn test was used with the Bonferroni correction.

## Results

Mature biofilms of *M. smegmatis* produced on nylon filters showed different behavior than planktonic cultures or freshly inoculated biofilm (Supplementary Figures S1 and S2). In both planktonic culture and freshly inoculated filter, growth was necessary and thus resulted in longer lag phases and overall slower processes. Here, the focus was on mature biofilms all of which released large amounts of heat as measured by calorimetry. Similarly, rapid changes in headspace oxygen and carbon dioxide concentration were observed. This confirms the appropriateness of both techniques for monitoring the metabolism and associated respiration rates of mycobacterial biofilms. In both cases, differences between fast-growing and slow-growing mycobacteria were much smaller than expected based on growth characteristics in liquid or solid media (where growth is up to 20x slower for slow-growing species). The maximum difference in peak heat flow was measured between *M. smegmatis* (2298 ± 181 µW) and *M. phlei* (792 ± 81 µW). Differences between slow-growing *M. tuberculosis* (990 ± 204 µW) and *M. bovis* (1580 ± 91 µW) were lower. Similarly, oxygen consumption and carbon dioxide production rates were only two times lower in *M. tuberculosis* compared to *M. smegmatis* (Table 1).

**Table 1:** Average heat production rate (P_10_, from IMC) and gas production or consumption rate (from TDLAS) during the first 10 hours. To compare IMC and TDLAS, theoretical heat flow values were recalculated from oxygen consumption rate and carbon dioxide production rate using Equation 1.

	*M. smegmatis*	*M. tuberculosis*	N (per organism)
P_10_ (heat flow average) (measured - mW)	1.60 ± 0.20	0.83 ± 0.20	4*
O_2_ consumption rate (measured – nMole/s)	3.3 ± 0.2	1.7 ± 0.3	4
P_10_ O_2_ (calculated - mW)	1.56 ± 0.08	0.81 ± 0.20	NA
CO_2_ production rate (measured – nMole/s)	2.4 ± 0.1	1.1 ± 0.3	4
P_10_ CO_2_ (calculated - mW)	1.29 ± 0.05	0.57 ± 0.01	NA

^*^7 replicates were measured for *M. tuberculosis*.

**Figure 2:**
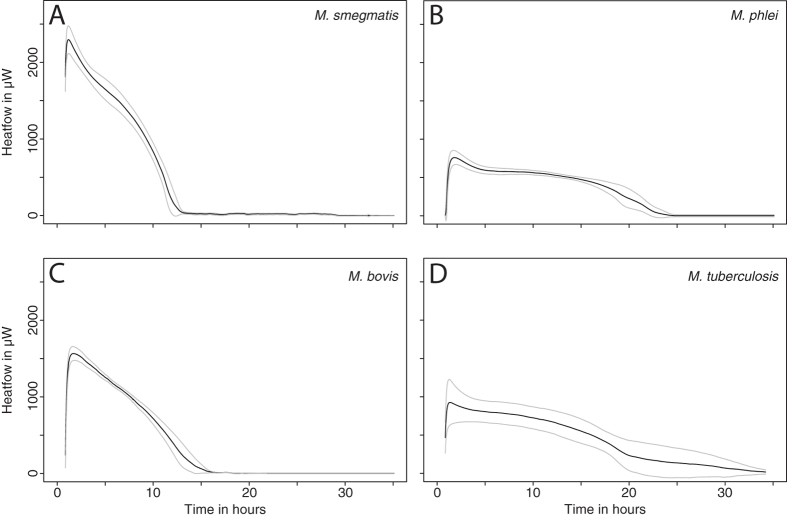
Metabolic heat production of the mycobacterial biofilms tested. (**A**) *M. smegmatis*, (**B**) *M. phlei*, (**C)** *M. bovis*, (**D)** *M. tuberculosis*. Black lines represent the average of 4 replicates and grey lines represent the upper and lower values of standard deviations. 7 replicates were performed for *M. tuberculosis*.

With respect to the evolution of the heat flow profile over time, as soon as mature mycobacteria biofilms were transferred on fresh 7H9 medium filled in 20 ml glass vials, heat flow rose very rapidly. A maximum metabolic heat production rate was reached within 1 hour for *M. smegmatis* and 2 hours for *M. phlei* and *M. bovis*, while *M. tuberculosis* required 3 hours to reach the maximum heat flow. After this period the metabolic heat production rate declined and returned to baseline within 15–40 hours. Overall, the shape of the heat-flow pattern was similar for all tested biofilms (a rapid rise to a maximum value within hours, followed by a slower decrease until no detectable metabolic heat production was observed) (Fig. 2). Differences in the metabolic activity for all tested mycobacteria reached a maximum of 62% compared to the average value. This corresponds to a roughly a two-fold difference between *Mycobacterium* strains, where a 20-fold difference would be expected based on conventionally grown cultures. The metabolic activity of biofilms therefore appears high, and within a small range for strains that have very different growth rates in conventional liquid or solid cultures. Similarly, all other parameters showed low variation between slow- and fast-growing mycobacteria (Supplementary Table S1).

The short lag phase (0.2–1.0 hour) and rapid rise in metabolic activity of the mycobacterial biofilms on 7H9 medium indicates that mycobacterial cells within the biofilm can rapidly reactivate when conditions become favorable, such as with transfer to a fresh medium. Similarly, *M. smegmatis* biofilms reach peak metabolic activity in shortest period of time (1.2 ± 0.0 hours) followed by *M. bovis* (1.7 ± 0.1 hours), *M. phlei* (1.8 ± 0.2 hours) and finally *M. tuberculosis* (3.2 ± 2.3 hours) (Supplementary Table S1).

**Figure 3:**
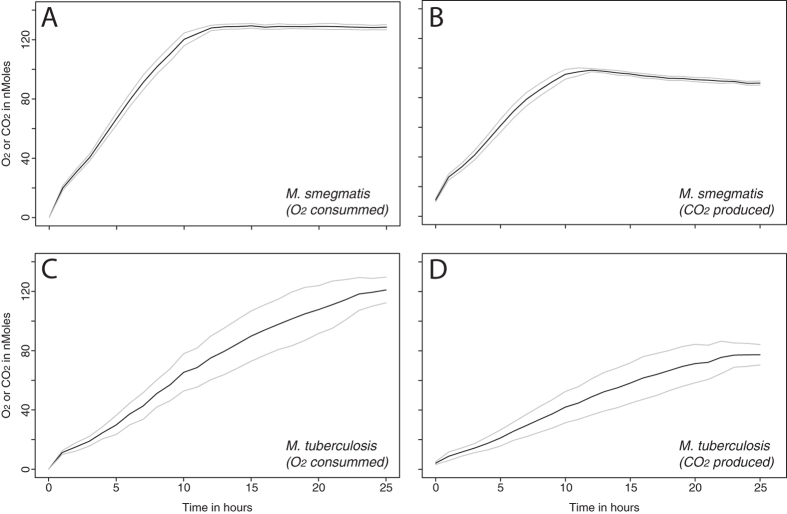
Oxygen consumption and carbon dioxide production by biofilms of *M. smegmatis* and *M. tuberculosis*. (**A**,**B**) Oxygen consumption and carbon dioxide production by biofilms of *M. smegmatis*. (**C**,**D**) Oxygen consumption and carbon dioxide production by biofilms of *M. tuberculosis*. Black lines represent the average of 4 replicates and grey lines represent the upper and lower values of standard deviations.

Gas composition in the headspace of glass vials containing mature *M. smegmatis* or *M. tuberculosis* biofilms was measured using TDLAS (Fig. 3). The amount of oxygen in the headspace declined from the very beginning of the experiment with a simultaneous increase in the amount of carbon dioxide. When no further changes in gas concentration could be observed, the experiment was terminated. Both mycobacterial biofilms consumed all available oxygen whereas the resulting carbon dioxide concentration in the headspace was between 12.5 ± 0.19% for *M. smegmatis* and 10.7 ± 0.97% for *M. tuberculosis*.

Considering the amount of oxygen consumed and carbon dioxide produced, the observed ratio is in agreement with the glycerol respiration equation (Equation 1). In contrast to the heat production rate, oxygen consumption and carbon dioxide production for *M. smegmatis* biofilms were twice as fast as for *M. tuberculosis* biofilms (Table 1). Oxygen was exhausted and carbon dioxide concentration in the headspace was stable after 14 hours for *M. smegmatis* biofilms, but took 25 hours for *M. tuberculosis* biofilms. Again, such differences are small compared to those commonly observed in planktonic or solid cultures.

Both IMC and TDLAS provide direct quantitative information about the metabolic activity of mature biofilms based on independent physicochemical measurements. Therefore, using Equation 1 and combining calorimetry and gas headspace measurements it is possible to compare the rates of metabolic heat emission (heat flow). Similarly, the total energy release (enthalpy change, ΔH) measured by IMC and calculated based on the amount of oxygen and carbon dioxide in vials can be compared. These calculations lead to closely related values for measured and calculated heat values using oxygen and carbon dioxide data (Table 2). The measured enthalpy by IMC and calculated enthalpy based on oxygen concentration in the vial headspace are in agreement.

**Table 2:** The enthalpy change measured by IMC and calculated based on the oxygen consumption and carbon dioxide production results.

	IMC ΔH, Joule (n = 4)	O_2_ ΔH, Joule (n = 4)	CO_2_ ΔH, Joule (n = 4)
		***Glycerol***	
*M. smegmatis*	−59.27	−60.53	−49.65
*M. tuberculosis*	−59.05	−57.22	−42.48
		***Glucose*** ^†^	
*M. smegmatis*		−59.79	−42.04
*M. tuberculosis*		−56.52	−35.96
		***Organic matter*** ^†^	
*M. smegmatis*		−58.24*	n.a.
*M. tuberculosis*		−55.05*	n.a.

^*^Thornton oxycaloric equivalent (i.e., 455 ± 15 KJ/mole O_2_) is used for those calculations.

^†^Provided for comparison purposes only.

TDLAS provides information about the metabolic activity of a microorganism based on oxygen availability, whereas IMC follows the entire metabolic process independent of gas concentration. As oxygen is the only terminal electron acceptor for obligate aerobe such as mycobacteria, it is expected that there would be agreement between IMC values and oxygen-based enthalpy calculations. For carbon dioxide, there is a slight difference in enthalpy measured using IMC and calculated based on carbon dioxide produced. Mature *M. smegmatis* and *M. tuberculosis* biofilms show a 16% and 28% difference in enthalpy calculations based on glycerol consumption. This difference fits within the range expected for maintenance energy of bacteria, which is between 10% and 31% of the energy expenditure (and thus heat release^34^). Slightly different values could be calculated assuming that glucose from the OADC supplement would be the primary energy source (28% and 39%, respectively). However, such values are still close to the expected differences in maintenance energy.

Taken together, these results show that differences between slow-growing and fast-growing mycobacterial biofilms are much smaller than expected based on their conventionally cultured counterparts. Only a short period of time is needed for these biofilms to recover high activity when appropriate conditions (nutrients or oxygen) become available. The determined parameters (metabolic heat production rate, oxygen consumption, carbon dioxide production, and enthalpy) retained similar values.

## Discussion

Biofilm cells differ from planktonic cells in many ways. Biofilms are known to protect pathogens from phagocytosis and to increase tolerance to drug treatment. Slower growth rates and lower metabolic activity are implicated in a higher tolerance to drug treatment. However, in some studies biofilms were shown to have higher metabolic activities compared to their planktonic counterparts^35^. In our study, the results of IMC and TDLAS measurements (two independent and non-invasive techniques) indicate that mature biofilms of slow-growing and fast-growing mycobacteria have comparable metabolic activities. Although differences in grawth and “activity” for slow- and fast-growing mycobacteria have been observed in previous studies using liquid or solid media, biofilms of these organisms show a much more similar profile. Additionally, only a short period of time is needed for such biofilms to recover full activity when transferred to appropriate conditions (such as fresh medium). This seems counter-intuitive, as available literature on doubling time for fast-growing species (*M. smegmatis* and *M. phlei*: 3.6–4.5 hours) notes values about 12 times shorter than their slow-growing counterparts (*M. tuberculosis*: 18–54 hours)^36, 37^. Using IMC data, the reactivation rate (or the heat growth rate μ_Q_ calculated using the same approach as for the growth rate) would give an activity-doubling time between 8.7–17.8 minutes. Such an unrealistic doubling time clearly emphasize that very little (if any) growth takes place in the mature biofilms, and the rise in activity mostly corresponds to inactive cells becoming metabolically active again once placed on fresh medium. The same reasoning can be applied to the time to reach peak activity, which is also very short and would barely allow for growth of one generation. Finally, the fact that about 70% of oxygen is converted into carbon dioxide for *M. smegmatis* while about 63% is converted for *M. tuberculosis* (Fig. 3) also supports the idea the very little carbon is incorporated into net biomass production. This is especially of note when considering that 10–31% of the energy is dedicated to maintenance, and not cell growth.

Based on oxygen consumption and carbon dioxide production rates it is also possible to estimate the theoretical heat flow for comparison with IMC heat flow from mature mycobacteria. Indeed, the maximum measured heat flow using IMC matches well with calculated heat flow for *M. smegmatis* and *M. tuberculosis* (Table 2). Furthermore, the calculated heat flow based on carbon dioxide production rate of both mature biofilms confirm the fact that 50 µmol s^−1^ CO_2_ lead to 5 µW heat production rate^38^.

It is known that the oxygen concentration in the biofilm decreases with the increasing depth of biofilm^23^. The results of IMC and TDLAS show a direct relationship between oxygen availability and the metabolic activity of mature mycobacterial biofilms, where oxygen depletion leads to a reduction in metabolic activity. The metabolic activity of *M. smegmatis* reached a minimum as soon as the available oxygen in the headspace of the glass vials was consumed (within 13–14 hours).

Two independent techniques demonstrated that there are only minor differences in metabolic activity between mature slow- and fast-growing mycobacterial biofilms. For example, *M. bovis*—a well-known, slow-growing mycobacteria—exhibited metabolic activity close to that observed for the fast-growing *M. smegmatis*. The same phenomena can be observed for slow-growing *M. tuberculosis*, for which metabolic activity is even higher than the fast-growing *M. phlei*. Further headspace gas production or consumption rate determined using TDLAS also verified the little difference in metabolic activity of mature *M. smegmatis* and *M. tuberculosis* biofilms occurred.

In this study, we investigated 4 different mycobacterial biofilms that belong to fast-growing and slow-growing *Mycobacterium* strains using IMC. In addition, to gain more information about the activity of two mycobacterial biofilms further investigation was performed by TDLAS. Our results suggest that there is a strong need to further explore the combination of these technique for the study of biofilm bioenergetics behavior. It could also be useful to investigate mature mycobacterial biofilms from the tissue of infected patients to gain more insight to their metabolic activity. For mature biofilms especially, where minimal growth is observed, the combination could be a very valuable tool to get more insights into maintenance energy requirements. Such an approach could also be useful for environmental mycobacterial biofilms such as those of *M. marinum* and *M. ulcerans* that can be found in aqueous environments such as rivers, aquariums, or hot tubs^39, 40^ and could potentially be transferred to humans. Additionally, biofilms that form on catheters or implants might be tested to find the most appropriate drug^41, 42^. Previous studies have also shown that mycobacteria biofilms formed in urine or artificial urine had a higher activity that was sustained for a longer period when compared to planktonic cells^35^, and breast implant infection by mycobacterial biofilms have been shown to be common in recent years^43–45^. The results of this study might also be considered for novel drug screening as antibiofilm compounds (or biofilm penetrating compounds) are of particular interest not only for mycobacteria but also for many other pathogens. Similarly, patient care could be improved, as infections accompanied by biofilm formation are difficult to treat and have deleterious effects on human health.

## Electronic supplementary material

Supplementary material
