# Supplementary material for: Metabolic activity of mature biofilms of Mycobacterium tuberculosis and other non-tuberculous mycobacteria
Source: Sci Rep. 2017 Aug 23;7:9225. doi: 10.1038/s41598-017-10019-4 (PMC5569076; doi:10.1038/s41598-017-10019-4)
Supplement: Supplementary file 1 — Supplementary material [file 41598_2017_10019_MOESM1_ESM.pdf]

Supplementary material

Metabolic activity of mature biofilms of *Mycobacterium tuberculosis* and other non-tuberculous mycobacteria

Anna Solokhina[1], David Brückner[1,2], Gernot Bonkat[3], Olivier Braissant[1]

1: Center of Biomechanics & Biocalorimetry, University Basel, Gewerbestr. 14, CH-4123 Allschwil, Switzerland.

2: F. Hoffmann – La Roche, Ltd., Sterile Drug Product Manufacturing, Wurmisweg, CH-4303 Kaiseraugst, Switzerland.

3: Alta Uro AG, Centralbahnplatz 6, CH-4051 Basel, Switzerland.

Supplementary material

Table S1: growth parameters determined by IMC. Pmax (maximum metabolic heat production rate), μ (heat growth rate λ (lag phase), Q (total heat produced)

	**Pmax (μW)**	**μ (growth rate) ($J \bullet h^{-1}$)**	**Time to peak (h)**	**λ (lag phase) (h)**	**Q (Heat) (J)**
M. smegmatis	2298 ± 181	7.2 ± 0.4	1.2 ± 0.0	0.2 ± 0.1	59.3 ± 4.8
M. phlei	792 ± 81	2.7 ± 0.1	1.8 ± 0.2	1.0 ± 0.3	40.4 ± 2.3
M. bovis	1580 ± 91	5.6 ± 0.2	1.7 ± 0.1	0.4 ± 0.1	46.2 ± 2.9
M. tuberculosis	990 ± 204	3.4 ± 0.7	3.2 ± 2.3	0.8 ± 0.4	51.4 ± 8.7

* (see main text for details)

Figure S1

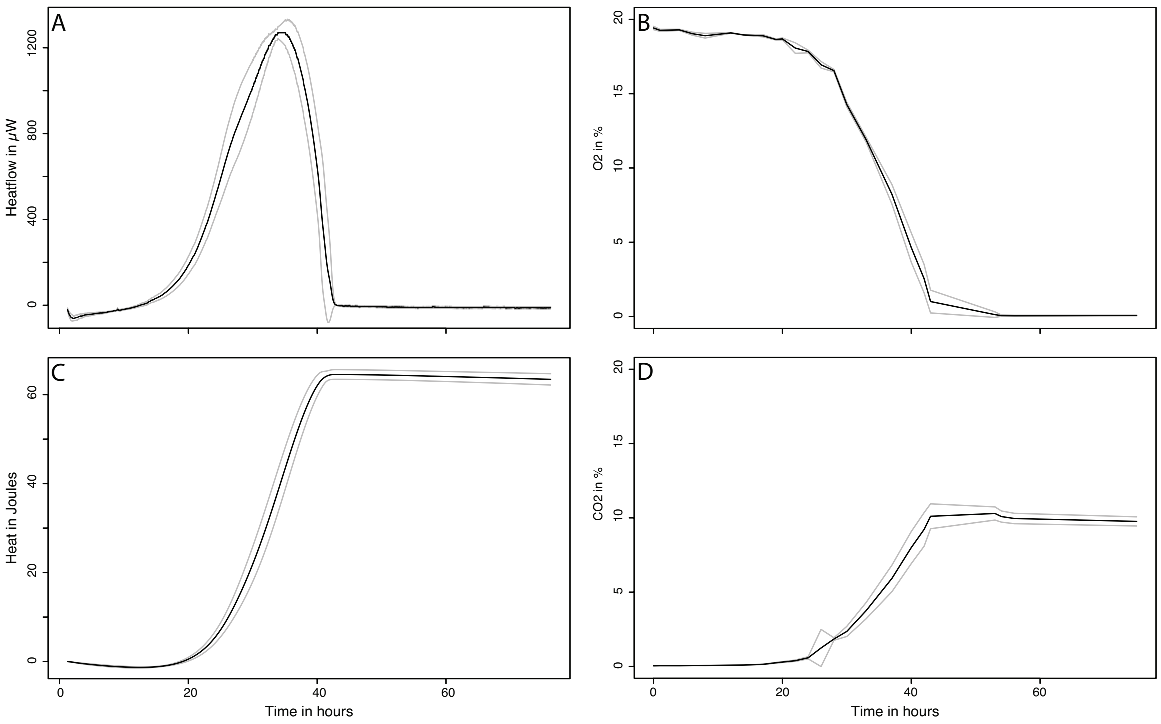

Figure S1: Calorimetric (A,C) and headspace gas composition (B,D) for vials containing a freshly inoculated nylon filter. A) Heat flow (metabolic heat production rate) from the growing biofilm, B) Oxygen consumption of the growing biofilm. C) Heat over time curve (area under the curve of A) for the growing biofilm. D) Carbon dioxide produced by the growing biofilm. Note that the growing biofilm (from freshly inoculated filters) has a longer lag phase and a lower activitz compared to its mature counterpart (see main text.).

Figure S2

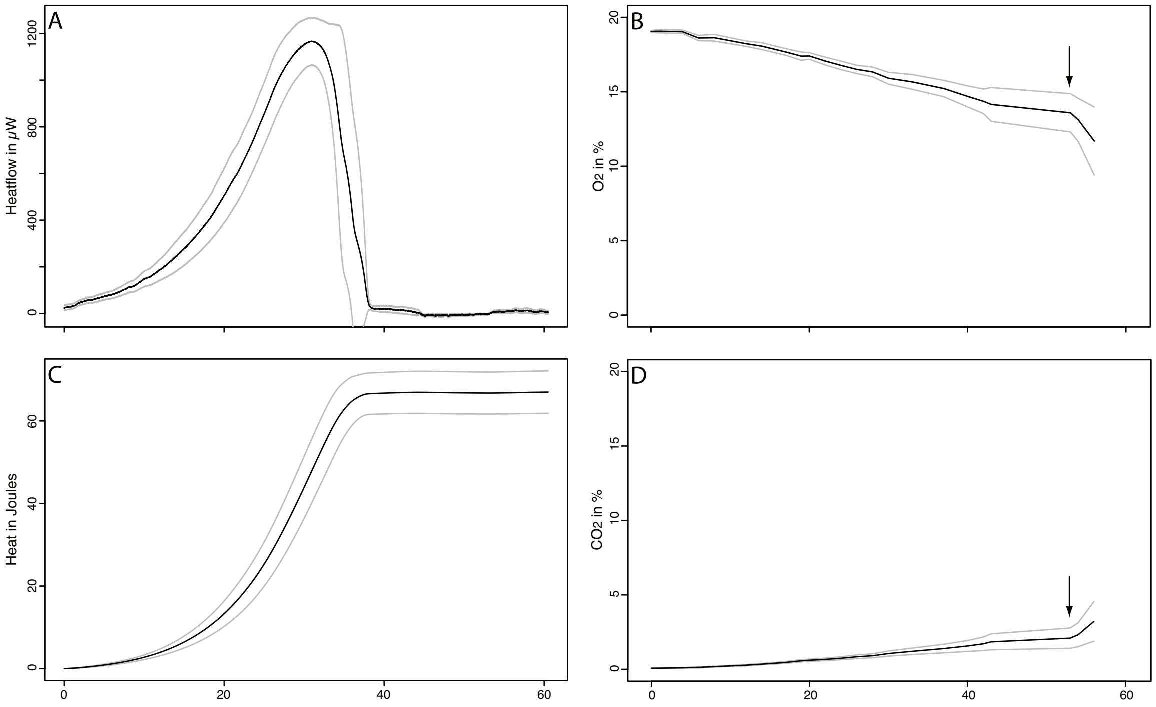

Figure S2: Calorimetric (A,C) and headspace gas composition (B,D) for vials containing a liquid culture of *M.smegmatis* without agitation. A) Heat flow (metabolic heat production rate) from the growing liquid culture, B) Oxygen consumption of the growing liquid culture. C) Heat over time curve (area under the curve of A) for the growing liquid culture. D) Carbon dioxide produced by the growing liquid culture. Note that for calorimetric data the growing liquid culture has a longer lag phase and a lower activity compared to its mature counterpart (see main text.). In addition for the *M.smegmatis* liquid culture headspace gas composition data (O2 consumption and CO2 production) show a disconnection compared to their biofilm counterpart. Indeed respiration rates are much lower although metabolic heat production rate remains similar to the growing biofilm (Fig. S1)
